# Investigation of serum beta-defensin-1 levels in bovine trichophytosis cases

**DOI:** 10.14202/vetworld.2021.2508-2511

**Published:** 2021-09-24

**Authors:** Aynur Simsek

**Affiliations:** Department of Internal Medicine, Faculty of Veterinary Medicine, Dicle University, 21280, Diyarbakir, Turkey.

**Keywords:** beta-defensin-1, bovine, serum, trichophytosis

## Abstract

**Background and Aim:**

Antimicrobial peptides are polypeptides that are a component of innate immunity and exhibit antifungal activity. This study aimed to investigate serum beta-defensin-1 levels in cattle diagnosed with trichophytosis, which is a zoonotic skin disease that affects several animal species.

**Materials and Methods:**

A total of 23 young cattle, aged 2-4 months, of different breeds and sexes were selected. Of these, 16 cattle were clinically diagnosed with trichophytosis and seven were healthy.

**Results:**

The mean serum beta-defensin-1 levels of the infected animals were lower than those of control animals, yet the difference between the two groups was not significant (p>0.05).

**Conclusion:**

No significant alterations occurred in serum beta-defensin-1 levels of cattle with trichophytosis.

## Introduction

Skin diseases that directly affect the quality of the hide, skin, fleece, and fur of animals cause major economic losses in the livestock industry. Bacteria, viruses, fungi, and parasites can cause dermatitis [1]. A fungal infection of the keratinized layer of the epidermis and hair follicles is referred to as ringworm or dermatophytosis [2-4]. Since *Trichophyton verrucosum* is the most common agent isolated from bovine dermatophytoses, cattle ringworm is also referred to as trichophytosis [2]. This disease, which is widespread in the tropical and subtropical regions of the world [1,5], mostly affects young and immunosuppressed animals [6]. Trichophytosis lesions, which are circular in shape, asbestos- or chalk powder-like in appearance and characterized by scaling, are generally observed around the eyes and on the forehead, cheeks, ears, and nose, but may spread to the neck, back and rump, and even the whole body in generalized cases [7-9]. The disease is transmitted through fungal spores or direct contact with infected animals or contaminated materials [10]. Once the infection develops, the intensity of the dermal response varies with the reaction of the host to the metabolic products of the fungus, virulence of the causative agent, localization of the infection, and environmental factors [11].

During the inflammatory process, keratinocytes and cellular components of the host defense system, including neutrophil leukocytes, mast cells, eosinophil leukocytes, and macrophages, participate in epidermal innate immunity [12]. Neutrophil leukocytes and monocytes, which are activated through inflammatory signals transduced by cytokines, chemokines, and complement components, migrate to the infection site. Then, as a result of various reactions, reactive oxygen mediators and antimicrobial peptides are produced and/or released, and fungi are killed or damaged.[13]. Antimicrobial peptides are polypeptides that participate in innate immune response and are secreted by polymorphonuclear leukocytes, macrophages, and mucosal epithelial cells [14]. Defensins, a major group of these peptides, are cationic molecules that are defined by the presence of three intramolecular disulfide bridges formed by six cysteine residues (C1-C5, C2-C4, and C3-C6), are composed of 30-40 amino acids, and have a molecular weight of 3.5-6 kDa [15]. Based on their size and pattern of disulfide bonding, defensins are classified into three subgroups, namely, alpha, beta, and theta defensins, which demonstrate antifungal, antibacterial, antiparasitic, and antiviral activities [16-18]. In addition to their antimicrobial effects, beta-defensins (b-defensins) are reported to mediate angiogenesis and play a role in epithelial growth and differentiation during wound healing [16]. β-defensins, which are secreted from several tissues in cattle, are also referred to as bovine neutrophil beta-defensins (BNBD) [19]. Studies have reported that following the development of skin infection, the level of antimicrobial peptides found in the skin increases because of neutrophil degranulation [20], and the production of β-defensin plays an important role in skin defense [21]. While increased β-defensin levels have been reported in the serum of humans with psoriasis [22,23] and the skin of dogs with atopic dermatitis [21], to the best of author’s knowledge, no report on serum β-defensin levels in cattle, either healthy or with dermal infection, has been published till date.

Thus, the present study aimed to determine the serum levels of β-defensin-1, in cases of bovine trichophytosis to help provide a better understanding of the pathogenesis, diagnosis, and treatment of this disease.

## Materials and Methods

### Ethical approval

Ethical approval was not required for this study as samples were collected for diagnosis and treatment purposes. Moreover, the study does not contain animal experimental trials.

### Study period and location

The samples were collected from March 2018 to June 2018. A total of 23 young cattle, including 16 animals diagnosed with trichophytosis and seven healthy animals, aged 2-4 months, were included in this study. Sick animals with skin lesions were diagnosed with trichophytosis based on the clinical symptoms manifested. The study was conducted in the Diyarbakir Province of Turkey.

### Sampling

Jugular blood samples were taken from the animals to determine serum β-defensin-1 levels, red blood cell (RBC) count, hemoglobin (HGB) concentration, hematocrit (HTC) value, white blood cell (WBC) count, and lymphocyte, monocyte, and granulocyte percentages. Serum β-defensin-1 levels were measured using a commercial enzyme-linked immunosorbent assay kit (SinoGeneClon Biotech Co., Ltd., Hanzhou, China). Blood analyses were performed using a Mindray BC 2800 hematology analyzer. All samples were processed at the laboratory of the Internal Medicine Department of Dicle University, Veterinary Faculty, Diyarbakir, Turkey.

### Statistical analysis

Data obtained in this study were statistically analyzed using theStatistical Package for the Social Sciences 16.0 Windows program (SPSS Inc., Chicago, IL, USA). The significance of differences between the two groups (infected and healthy groups) was determined using independent t-test. Values are presented as mean±standard error of mean. p<0.05 was considered statistically significant.

## Results

**Figure-1:**
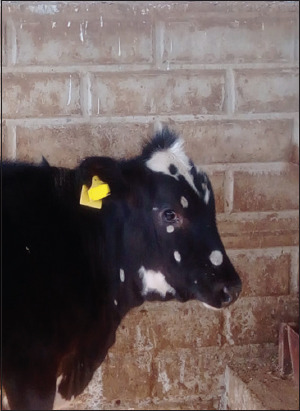
Skin lesions on the head and neck.

**Figure-2:**
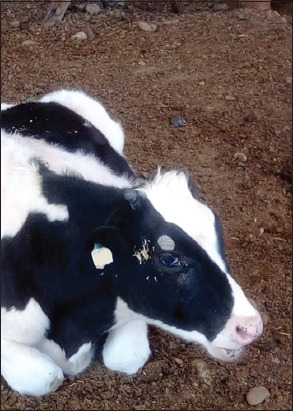
Skin lesions around the eyes.

The animals, which were diagnosed with trichophytosis, displayed grayish-white, asbestos-like, thick-crusted circular lesions in the head-and-neck areas (Figures-1 and 2).

In comparison with the control group, cattle in the infected group had higher RBC and WBC counts, HGB concentrations, HTC values, and granulocyte percentages but lower lymphocyte and monocyte percentages. Differences between the two groups for RBC counts (p<0.01), HGB concentrations (p<0.05), HTC values (p<0.05), and lymphocyte percentages (p<0.05) were statistically significant (Table-1).

**Table-1:** Hematological parameters of the infected group and control group.

Parameters	Control group (n=7)	Infected group (n=16)	p-value
RBC (×10^6^/mm^3^)	8.31±0.21	9.82±0.28	0.00
HGB (gr/dl)	9.67±0.34	10.89±0.31	0.03
HTC (%)	28.90±1.02	32.23±0.88	0.04
WBC (×10^3^/mm^3^)	7.00±0.47	9.12±0.74	0.09
Lymphocyte (%)	49.59±2.66	37.82±3.51	0.04
Monocyte (%)	12.69±0.62	12.66±0.87	0.98
Granulocyte (%)	37.73±2.63	49.40±3.55	0.05

n=Number, RBC=Red blood cell, HGB=Hemoglobin, HTC=Hematocrit, WBC=White blood cell

The mean serum β-defensin-1 levels of the infected group were lower than those of the control group, yet the difference was not significant (p>0.05) (Table-2).

**Table-2:** Serum β-defensin-1 levels of the infected group and control group.

Parameter	Control group (n=7)	Infected group (n=16)	p-value
β-defensin-1 (ng/mL)	1.41±0.38	1.27±0.28	0.07

n=Number

## Discussion

Dermatophytosis caused by *T. verrucosum* is a zoonotic skin disease that affects several animal species [9]. Host response to this disease, which is reported to be observed mostly in young animals, is mediated by innate and acquired immunities, and host protection requires the induction of a cell-mediated immune response [2].

Polymorphonuclear leukocytes and macrophages are primary phagocytic cells involved in the engulfment and degradation of fungal pathogens. Polymorphonuclear leukocytes, which have strong fungicidal mechanisms, migrate to the inflammation site and contribute to host defense [24]. In the present study, higher WBC counts and granulocyte percentages in the infected group demonstrate the important role of polymorphonuclear cells in host defense against trichophytosis.

A previous study reported that β-defensin-1 is produced by bovine neutrophils [25]. Panyutich *et al*. [26] suggested that the plasma defensin level could reflect the level of neutrophil leukocyte activation at the site of infection.

The previous studies in humans have shown that serum β-defensin levels increase in cases of lung cancer [27], diffuse panbronchiolitis [28], and cirrhosis [29]. Jaradat *et al*. [30] revealed increased serum β-defensin-2 levels in patients with *Trichophyton rubrum* infection. Furthermore, Tsybikov *et al*. [31] detected increased plasma α-defensin levels in patients with atopic dermatitis during exacerbation of the disease and reported that the clinical severity of the disease was positively correlated with the plasma α-defensin level.

Veldhuizen *et al*. [32] reported that the intestinal expression of β-defensin-1 and β-defensin-2 did not alter in pigs infected with *Salmonella* Typhimurium. Wang et al. [19] determined high levels of BNBD-1 and BNBD-2 in fresh bovine colostrum, with 0.45-9.36 µg/mL and 45.97-458.82 µg/mL, respectively, on postpartum day 2 and 0.49-7.19 µg/mL and 37.06-465.90 µg/mL, respectively, on postpartum day 5. Van Damme *et al*. [21] indicated that the gene expression of β-defensin-1 was high in dogs with atopic dermatitis.

In the present study, the mean serum level of β-defensin-1 of cattle with trichophytosis was 1.27±0.28 ng/mL and was lower than that of cattle in the control group, yet the difference between the two groups was not statistically significant. Since no previous literature report is available on serum β-defensin-1 levels in animals, a comparative evaluation of the results obtained in the present study could not be made.

## Conclusion

The serum β-defensin-1 levels of cattle with trichophytosis were lower than those of healthy control cattle. Thus, measurement of the tissue levels of β-defensin-1, in addition to the serum level, would be more meaningful.

## Authors’ Contributions

AS: Performed clinical examination of the animals, collected blood samples, analyzed and interpreted the data, designed and revised the manuscript. The author read and approved the final version of the manuscript.
